# Productivity of two *Brassica* oilseed crops in a Mediterranean environment and assessment of the qualitative characteristics of raw materials for bioenergy purposes

**DOI:** 10.1016/j.heliyon.2024.e26818

**Published:** 2024-02-23

**Authors:** Mario Licata, Davide Farruggia, Giuseppe Di Miceli, Francesco Salamone, Nicolò Iacuzzi, Teresa Tuttolomondo

**Affiliations:** a Department of Agricultural, Food and Forest Sciences, Università Degli Studi di Palermo, Viale Delle Scienze 13, Building 4, 90128, Palermo, Italy; b Research Consortium for the Development of Innovative Agro-Environmental Systems, Via Della Libertà 203, 90143, Palermo, Italy

**Keywords:** Brassica napus, Brassica carinata, Pure vegetable oil, Defatted seed meal, Crop residue, Combined heat and power system

## Abstract

Rapeseed (*Brassica napus* var. *oleifera* D.C.) and Ethiopian mustard (*Brassica carinata* A. Braun) are promising industrial crops for cultivation in the Southern Mediterranean area due to profitable yields under semi-arid conditions. The exploitation of raw materials produced by these crops is very convenient for farmers to produce bioenergy directly on-farm and permits them to create a short agri-energy supply chain. The purpose of this study was to determine their yield performance under rainfed conditions and make an economic assessment of a combined heat and power plant (CHP) system operating on pure vegetable oil (PVO). Tests were conducted in Sicily (Italy) from 2012 to 2014. Seed and crop residue yields were detected. The analysis of seed, defatted seed meal and crop residue, and the chemical-physical aspects of PVO were carried out according to conventional protocols. A pilot CHP system was used for cogenerating electricity and heat. In general, rapeseed had the highest seed (2.27 t ha^−1^) and oil (1.11 t ha^−1^) yields. The average oil content ranged from 44.88 % (Ethiopian mustard) to 45.73 % dry matter (rapeseed). Ethiopian mustard performed better than rapeseed in terms of aboveground biomass yield (5.49 t ha^−1^), in both years. The two crops showed different fatty acid profiles of the oil mainly due to diverse content of erucic and oleic acids. The CHP system had an average consumption of 14.41 kg PVO h^−1^. These results confirm that the productivity of the species can be appreciable in the Southern Mediterranean area and indicate the use of raw materials of these crops as crucial to the development a sustainable short agri-energy supply chain.

## Highlights

• Rapeseed and Ethiopian mustard provided different yields.
• The exploitation of raw materials is crucial into a short agri-energy chain.
• Pure vegetable oil allows the farmers to become bioenergy producers.
• Combined heat and power system permits to obtain a sequence of net benefits.
• Government incentives is fundamental to exploit the pure vegetable oil as a biofuel.

## Graphical abstract

**Figure:**
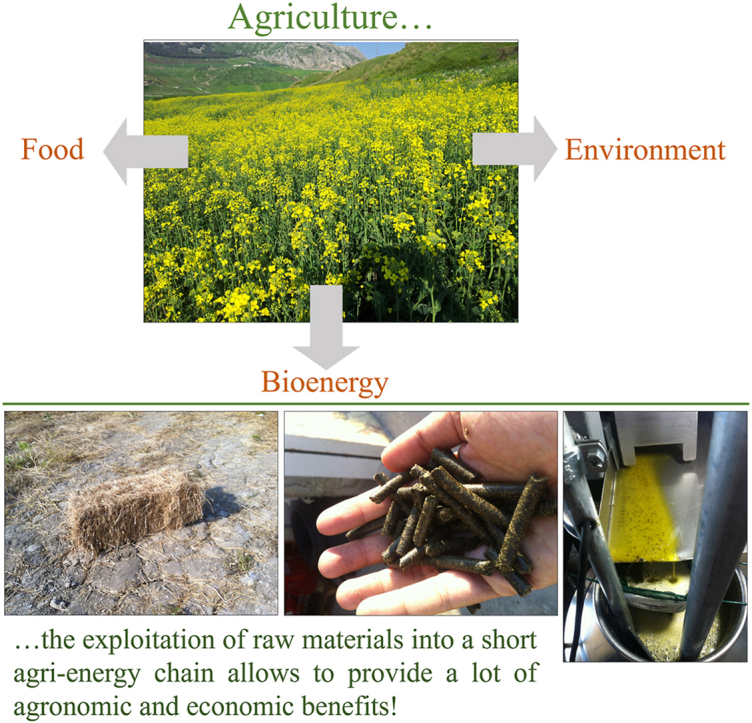

## Abbreviations

ADF: Acid detergent fiber
ADL: Acid detergent lignin
ANOVA: Analysis of variance
AOAC: Association of the Official Analytical Chemists
ASTM: American Society for Testing Materials
CHP: Combined heat and power
DM: Dry matter
DSM: Defatted seed meal
FPT: Fiat Powertrain Technologies
GCV: Gross calorific value
GDDs: Growing degree days
GL: Glucosinolate
IRR: Internal rate of return
NDF: Neutral detergent fiber
NMR: Nuclear Magnetic Resonance
NPV: Net present value
PVO: Pure vegetable oil
TSW: Thousand seed weight.

## 1 Introduction

Energy crops can offer new market opportunities and provide high added-value agricultural feedstocks which can be used to obtain bioenergy. Farmers growing these crops can play a relevant part in the European agri-energy market contributing to carbon neutrality by 2050, as the European Green Deal [1] aims to achieve. As stated by various authors [[2], [3], [4]], farmers can become both producers and end-users of bioenergy, creating a short agri-energy (agriculture-energy) supply chain. The concept of short agri-energy supply chain has been defined as a development model based on the close interaction between local agricultural production and its energy valorisation, which involves a limited number of economic operators and maintains geographical and social relations between energy producers and consumers [5]. the farmers can consume the self-produced energy directly on-farm and/or sell it externally, increasing greatly their income [6]. However, this scenario requires significant changes to agricultural systems and new business models for farmers.

Nowadays, in Southern Europe, growing energy-intensive crops fall short of the potential benefits they can provide for various reasons. Factors affecting the distribution of energy crops include the lack of a necessary market, the adoption of different cultivation practices with respect to those required by traditional food crops and higher land costs [7]. Furthermore, the public debate that energy crops may replace food crops and negatively affect biodiversity conservation in agriculture is relatively widespread, especially when the crops are grown on fertile land [8]. To reverse this trend, it is necessary to provide policies to support farmers who decide to choose multifunctional agriculture and adopt good practices in farming energy crops on different types of agricultural land.

It is reasonable to assume that, in southern European environments, oilseed crops offer a realistic opportunity for farmers as starter rotation crops, they can improve soil fertility and be included in rotations with winter cereals without replacing food production. In Italy, many authors [[9], [10], [11], [12]] have demonstrated previously that rapeseed (*Brassica napus* L. var. *oleifera* D.C.) and Ethiopian mustard (*Brassica carinata* A. Braun) are adapted to central-southern areas and allow the farmers to obtain valuable yields and diversify them as well. Interest in rapeseed and Ethiopian mustard is witnessed by numerous Italian studies concerning the bioenergy [[13], [14], [15]]. Given the richness in erucic and linoleic acids, the oil is mainly exploited for biodiesel production by transesterification reaction [[16], [17], [18], [19]]. As well known, biodiesel is one of the most popular biofuels in the world and represents an alternative to fossil fuels. Biodiesel production on a small farm-scale, usually requires cultivation practices with high energy inputs during the crop growth stages, to which the cost of oil transformation must be added [20]. As consequence, total costs of the biodiesel production both in economic and environmental terms can be very high [21] and often unsustainable for farmers, especially when agricultural feedstocks are not sufficiently valorised [3,9,22]. In this scenario, the exploitation of all raw materials produced by oilseed crops, including pure vegetable oil (PVO), defatted seed meal (DSM) and crop residues, can also contribute to bioenergy production.

As stated by Russo et al. [23], the direct use of PVO as a biofuel seems to be of interest due to favorable comparison with biodiesel, although this practice is still marginal. PVO can be obtained directly on farm using easy pressing and filtering technologies [3]. The direct feeding of PVO into an energy cogeneration plant makes this biofuel a widespread energy source in the local area. Unlike biodiesel, PVO does not require the use of large quantities of water during the purification process [24] and a by-product of the production process is an oil-rich flour suitable for use in animal fattening. In addition, as reported by previous studies carried out in Italy [2,[24], [25], [26], [27]], DSM can be used in various productive sectors including the energy sector. On a final note, crop residues, such as straw, can also be exploited for energy production through the combustion process [28,29] or used as a substrate for biogas production [30,31] directly on farm.

This paper provides results of two-year tests on productivity of rapeseed and Ethiopian mustard in a Mediterranean environment. The main goals were: i) to determine the agronomic performance of two *Brassica* species under rainfed condition; ii) to assess the fatty acid composition of the oil and the chemical-physical properties of the raw materials; iii) to make an economic assessment of a pilot scale combined heat and power (CHP) plant working on PVO during two different periods.

## 2 Materials and methods

### 2.1 Test site

Trials were conducted on a cereal livestock farm in Castronovo di Sicilia, in the Western Sicily (Italy) (37°67′41″ N - 13°56′52″ E, 570 m a.s.l.) during the growing seasons of 2012–2013 and 2013–2014. The climate of the study site is warm and temperate with dry summers [32]. With reference to time series 2000–2020, the annual rainfall is 550 mm and the annual temperature is 14 °C, on average. The soil is heavy clay, and classified as Typic and/or Vertic Xerochrepts accordingly to the United States Department of Agriculture (USDA).

### 2.2 Weather data

Rainfall and temperature data were provided by a meteorological station of the Sicilian Agro-Meteorological Information Service (2022) [33]. It was situated about 10 km from the farm and equipped with various sensors to take measurements of the main climatic parameters. In the present study, daily minimum and maximum air temperature and rainfall data were recorded using a data logger (model WST1800).

### 2.3 Main cultivation practices

An experimental field was set up for each species. PR46W14 hybrid of *Brassica napus* and ISCI 7 selection of *Brassica carinata* were used for the trials. In each field, experimental plots were arranged in a randomized complete block design [34] with three replications during the two growing seasons. Each plot measured 15 m^2^ (5 m × 3 m). *Triticum durum* Desf. was the preceding crop. The fields were managed with conventional tillage and mineral fertilisation. Soil was ploughed at a depth of 35 cm and harrowed. Sowing occurred on November 5, 2012 and on November 8, 2013, adopting a density of 75–80 viable seeds m^−2^ and row spacing of 15 cm. The seeding rate was based on the purity and germination rates found on the seed. A seed drill was used to position the seeds at equal distances and proper depth (2–3 cm), ensuring they get covered with soil. It was divided into two compartments, one for seed and another for fertiliser distribution. At sowing time, 220 kg ha^−1^ of phosphorus fertilizer (simple super-phosphate) was applied. 150 kg ha^−1^ of nitrogen fertiliser was used, 50 kg ha^−1^ (ammonium sulphate) at rosette growth stage and 100 kg ha^−1^ (ammonium nitrate) prior to stem elongation. The post-emergence fertilisation of the plots was carried out by a centrifugal fertiliser spreader. Plants were cultivated under rainfed condition. Post-emergence weed control was carried out. Dicotyledonous weeds were mechanically managed while metazaclor (2.0 l ha^−1^, at the second leaf stage) and fluazifop-*p*-butyl 13:40% (1.0 l ha^−1^, at stem elongation stage) were applied by a portable sprayer with an operating pressure of 250 kPa against graminaceous weeds. Regarding pest control, imidacloprid at a rate of 2 l ha^−1^ at flowering stage was exploited against *Meligethes aeneus* F. by using the same machine. A combine harvester with a wheat-cutting bar operated in the 1st 10-day period of July during growing seasons 2012–2013 and 2013–2014. When the seed moisture level was less than 14%, harvesting was made.

### 2.4 Plant growth and measurements

The main growth stages of rapeseed and Ethiopian mustard were determined according to Lancashire et al. [35]. The germination stage (BBCH scale code = 00–09) was measured from the sowing of the dry seed to the emergence of cotyledons. Leaf development (BBCH scale code = 10–19) was observed from the time the cotyledons unfold completely to nine or more leaves unfolded. Stem elongation stage (BBCH scale code = 30–39) was recorded from the beginning of stem elongation to more visibly expanded internodes. Flowering stage (BBCH scale code = 60–69) was detected from the beginning to the end of flowering. The development of seed (BBCH scale code = 70–79) was determined from 0% of pods reaching final size to nearly all pods reaching final size. The ripening stage (BBCH scale code = 80–89) was recorded until fully ripening (nearly all pods ripe, seeds dark and hard). Senescence stage (BBCH scale code = 97–99) was measured from the time of the plants becoming dry to the harvested product.

The detection of crop phenology was also based on calculation of accumulated growing degree days (GDDs). For each phenological stage, daily GDDs were calculated using a base temperature value of 4 °C [36] as follows [37]:

(1) $$GDD=\frac{\left(T_{\max}+T_{\min}\right)}{2}\;–\;T_{base}$$

Where: T_max_ and T_min_ are daily maximum and minimum air temperatures and T_base_ is the base temperature below which development ceases.

Seed and crop residue yields were estimated at harvesting stage on a area of 10 m^2^.

Plant height, number of siliques per plant, silique length, number of seeds per silique and 1000-seed weight (TSW) were detected on a sample of 10 plants per plot. Samples of each vegetable fraction of plant (leaves, stems, seeds and siliques) were dried at 60 °C in an oven (until constant weight) to determine the dry aboveground biomass. Subsequently, seeds were cleaned, dried, and crushed to obtain pure vegetable oil (PVO) and defatted seed meal (DSM).

A seed crusher machine (Bracco Company model Elle. Gi type 0.90) of 2.2 kW electric power with a capacity of 30 kg h^−1^ seeds was used for the test. A pilot scale CHP plant powered by Fiat Powertrain Technologies (FPT) with a nominal power of 75 kW h^−1^ was used for the cogeneration of electricity and heat, operating on PVO produced during the study. An average consumption of 14.4 kg PVO h^−1^ was estimated for this machine. The CHP plant was installed on a small farm, located in Campofelice di Roccella in the Western Sicily (37°59′33″ N - 13°52′35″ E, 8 m a.s.l.).

**Fig. 1:**
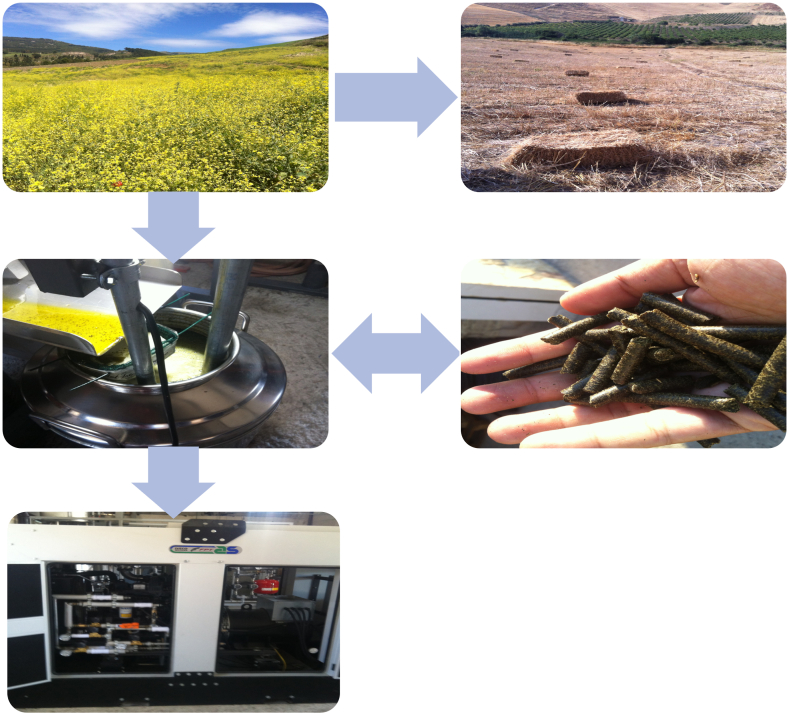
Main outputs of the agri-energy chain.

In Fig. 1, the main outputs of the agro-energy supply chain, including the Brassicas experimental fields, the seed crusher machine and the pilot scale CHP plant, are shown.

### 2.5 Analysis of seed, defatted seed meal and crop residue

Three samples of crop residue, DSM, and seed per plot were analysed to identify the chemical-physical properties. A composite sample per plot (500 g) was formed by combination and mixing all the three samples taken from the plot.

An elemental analyzer LECO CHN TruSpec was used to obtain by dry combustion the total content of carbon (C), hydrogen (H) and nitrogen (N) of seed, DSM and crop residue accordingly to the American Society for Testing Materials (ASTM D5373). Glucosinolate (GL) content of both the seeds and DSM was determined following the ISO 9167-1 method [38] with some minor modifications and changing the extraction procedure as Lazzeri et al. claimed [39]. The protein content was calculated from nitrogen using the standard factor of 6.25 and was reported as a percentage of dry matter (DM) [40]. Moisture content of seeds was obtained by oven-drying them at 105 °C for 12 h and calculated as the difference between the seed weights before and after this procedure, following the AOAC method [41]. Hexane (1.5:20, w/v) and *trans*-methylated with 2NKOH methanolic solution were used to extract fatty acids from ground seed, as reported by Conte et al. [42]. A gas chromatography-FID (Carlo Erba HRGC 5300 MEGA SERIES) equipped with a capillary column Restek RT x 2330 (30 m × 0.25 mm x 0.2 mm) was employed to analyse the fatty acid composition, following the internal normalization method [43]. Oil content was evaluated according to UNI EN ISO 5511:1998 [44]; a Nuclear Magnetic Resonance (NMR) technique by an MQC benchtop NMR analyser (Oxford Instruments) was used. Following the European standard method UNI EN 14775:2010, ashes content of PVO and crop residue were assessed by dry ashing the samples for 5 h at 550 °C in a muffle furnace. Following the Van Soest method [45], a FIWE fiber analyzer (Velp Scientifica) was employed to evaluate the neutral detergent fiber (NDF), acid detergent fiber (ADF) and acid detergent lignin (ADL) contents of crop residue. Gross calorific value (GCV) of crop residue and DSM was measured using a Berthelot-Mahler bomb calorimeter, accordingly to UNI CEN/TS 14918:2005 [46]. The glucide content of crop residue was obtained following the AOAC method [47].

### 2.6 Chemical-physical aspects of pure vegetable oil

The main chemical-physical aspects of the PVO examined were: oil density, oil kinematic viscosity, oil acidity and iodine value. Oil density at 15 °C was determined by an oscillation U-tube densitometer in accordance with the ISO 12185–1996/Cor.1:2001(E) method [48]. Oil kinematic viscosity at 40 °C was determined measuring the flow time of a predefined volume of a fluid affected by gravity through a calibrated glass capillary in accordance with UNI EN ISO 3104:2004 method [49]. The evaluation of the free fatty acids by means of volumetric titration by phenolphthalein permitted the determination of the oil acidity that was expressed as oleic acid in accordance with the UNI EN ISO 660:2009 method [50]. The iodine value was calculated using the UNI EN ISO 3961:2012 method [51] as follows: a test portion of oil was dissolved in solvent and Wijs reagent was added. After a certain time, potassium iodide and water were added, and titration of the liberated iodine with sodium thiosulfate solution was carried out.

### 2.7 Economic aspects

In Italy, various Decrees have provided economic incentives for promoting energy efficiency and the energy production from renewable sources, such as biofuels. These incentives can represent a tool aimed at creating more favorable conditions for increasing bioenergy production in energy supply chains. By exploiting these incentives, farmers can get an income for electricity production from CHP plant for a period of 20 years. Incentives for the production of bioenergy from PVOs using a pilot CHP plant were calculated in accordance with Italian Decree July 06, 2012 (2012–2014) and Italian Decree June 23, 2016 (2023). In both Decrees, the all-in incentive was calculated taking into consideration the lower of the two values: production of net energy by the combined heat and power system and energy released into the network. The difference between gross energy production and energy consumption from different activities was used to estimate net energy production. Consumption was fixed at 8 % of gross energy production as the CHP system operated on liquid biofuels with a capacity below 1 MW. The net energy produced by the CHP plant was then calculated as 55.21 kW h^−1^. The all-inclusive feed-in incentive (To) was calculated using the equation: To = Tb + Pr, where: Tb is the base feed-in incentive; Pr is the total premium.

With regards to the analysis of the financial benefits versus cost of the CHP plant, a model based on cash-flow was used. This method allows us to evaluate a number of parameters, such as the net present value (NPV), the internal rate of return (IRR) and the payback period. The model was prepared as a sequence of Net Benefits (NB) and, for each J-th year within the service life of the CHP plant, provided the difference between them: NB (J) = revenues (J) - expenditures (J) (€ year^−1^). The main revenue sources examined were: return from the production of electricity and thermal energy, sale of DSM. The main expenses considered in the model were: production costs of the PVOs, machinery costs, ordinary and extra-ordinary maintenance costs of the CHP plant. The return from the production of electricity was calculated multiplying the all-inclusive feed-in incentive by the operational hours of the CHP plant per year, considering a 20-year period. The return from the production of thermal energy was estimated as electricity not purchased from the Italian Electricity Grid in the same period. The sale price of DSM was estimated using data from the literature and partly collected on site. The production costs of the PVOs were estimated using the current market prices at the time of the study. The ordinary and extra-ordinary maintenance costs of the CHP plant were determined taking into consideration the prices reported in the manufacturer's use and maintenance manual and referred to 20-year period.

### 2.8 Statistical analysis

Data were submitted to the mixed effects model analysis of variance (ANOVA), using the software MINITAB 19 for Windows. Year and replicate were inserted as random factors. A *p*-value <0.05 was considered statistically significant. All values were showed using mean ± standard deviation calculations.

## 3 Results

### 3.1 Analysis of rainfall and temperature in the study area

**Fig. 2:**
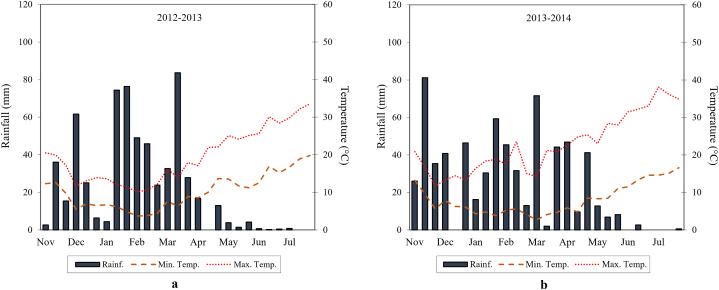
Rainfall and temperature trends during the 2012–2013 (a) and 2013–2014 (b).

Fig. 2 (a) and Fig. 2 (b) show the rainfall and temperatures trends during the 2012–2013 and 2013–2014 growing seasons.

Total rainfall during the first growing season was 606 mm, rather different from that of the second growing season (672 mm). The distribution of rainfall was prevalent in December and April. Between May and July, during crop ripening and senescence stages, rainfall was below 15 mm in both growing seasons. Greater rainfall distribution during winter and spring led to an increase in soil water availability that was exploited by crops during summer thereby affecting the productivity. In particular, the most relevant rainfall event occurred in the second 10-day period of March 2013 (84 mm). In both growing seasons, minima and maxima temperatures trends were similar and consistent with the ten-year average temperature. Decrease in temperature was observed from November to February while a continuous increase was recorded from beginning of March up to June, when ripening occurred. The highest maximum temperature value (38.1 °C) was detected in the first 10-day period of July 2014, when harvest occurred, whilst the lowest minimum temperature value (3.7 °C) was recorded in the first 10-day period of February 2013, when leaf development began.

In each growing season, the minimum temperatures fell below 4 °C during winter, but nevertheless the plants did not reveal any sign of frost damage. Moreover, the plants did not show signs of heat stress in summer, highlighting their great resilience to drought.

### 3.2 Plant growth stages

The 2-year average growth cycle length of rapeseed PR46W14 and Ethiopian mustard ISCI 7 was 232 and 248 days, respectively (Table 1).

**Table 1:** Duration and cumulative growing degree days required from various growth stages of *Brassica* species during two consecutive growing seasons. Average data are shown.

**Species**	**Duration (days)**						
	Germination	Leaf development	Stem elongation	Flowering	Development of seed	Ripening	Senescence
*B. napus* PR46W14	23	89	124	156	195	221	232
*B. carinata* ISCI 7	25	94	128	166	206	237	248
	**GDD (°C day)**						
	Germination	Leaf development	Stem elongation	Flowering	Development of seed	Ripening	Senescence
*B. napus* PR46W14	229	674	907	1188	1671	2070	2271
*B. carinata* ISCI 7	241	706	940	1299	1824	2373	2574

The growth cycle of the two crops was shorter in the first growing season compared to the second growing season. This can be explained by considering the amount of rainfall recorded during spring. In the 2012–2013 growing season, rainfall between March and May was 151 mm, lower than the 174 mm found in the same period during the second growing season. In the first growing season, the rise in air temperature and the lowest distribution of rainfall increased stress conditions causing early maturity in Ethiopian mustard and rapeseed plants.

During the study years, the earliest crop was rapeseed in each growth stage. Germination stage occurred on average within 23 days and small differences were found between the years depending on air temperature. Stem elongation stage was detected on average within 124 days from sowing date for rapeseed and within 128 days for Ethiopian mustard during the two years. Regarding the flowering stage, it was affected by rainfall and air temperature levels. In both years, the flowering stage started later in Ethiopian mustard than rapeseed plants. The duration of the flowering stage greatly influenced the development siliques and the subsequently ripening stage. Rapeseed plants reached fruit ripening stage earlier than Ethiopian mustard plants, on average. Senescence stage was observed when maximum air temperature was higher than 30 °C and no rainfall was recorded. The growth cycle ended before the 1st 10-day period of July in the two growing seasons for both the species.

The two crops accumulated different GDDs in the two growing seasons varying the minimum and maximum temperatures. An average of 2271 GDDs for rapeseed and 2574 GDDs for Ethiopian mustard were needed to complete the growth cycle. In general, at each growth stage, Ethiopian mustard accumulated the highest average GDDs.

### 3.3 Morphological and yield component performance

Results of ANOVA highlighted that the two random factors did not produce any significant variation for all morphological and yield parameters for both the species. Ethiopian mustard and rapeseed showed homogeneity as regards for silique length and TSW, whilst substantial variability was observed for plant height, number of siliques per plant and number of seeds per silique (Table 2).

**Table 2:** Morphological and productive parameters of rapeseed and Ethiopian mustard selections during two consecutive growing seasons. Average ± standard deviation values are shown (*n = 20*).

**Species**		**Plant height (cm)**	**Silique lenght (cm)**	**Number silique plant^-1^ (n)**	**Number seed silique^-1^ (n)**	**TSW (g)**
*B. napus* PR46W14		138.72 ± 1.59	6.26 ± 0.05	164.44 ± 4.94	25.55 ± 1.38	3.61 ± 0.05
	*Significance*					
	Year	n.s.				
	Replicate	n.s.				
*B. carinata* ISCI 7		157.94 ± 1.45	5.49 ± 0.16	321.63 ± 4.47	16.17 ± 0.78	3.40 ± 0.08
	*Significance*					
	Year	n.s.				
	Replicate	n.s.				

TSW: thousand seed weight.

n.s.: not significant.

**Fig. 3:**
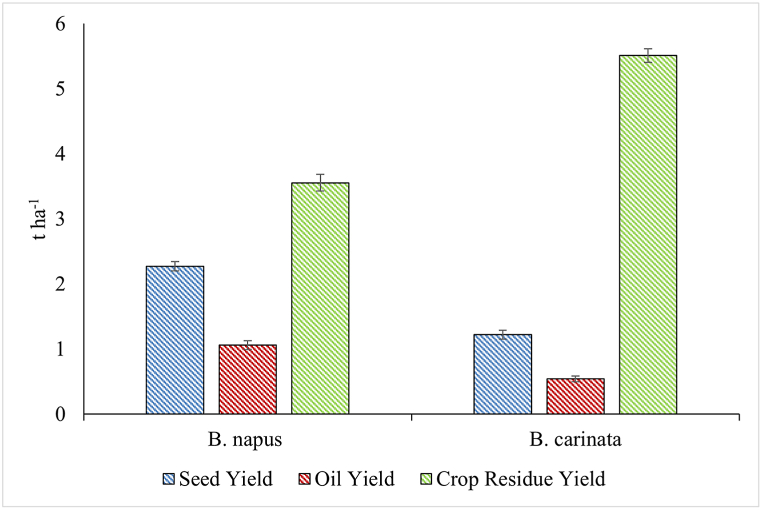
Seed, oil and crop residue yields of rapeseed and Ethiopian mustard selections. Average ± standard deviation values are shown (*n = 3*).

Ethiopian mustard had the highest values of plant height and number silique per plant, on average. Rapeseed obtained the highest average number of seed per silique and TSW value. In both growing seasons, seed losses were observed in the two *Brassica* experimental field but they were lower than 7 % and did not greatly affect the yield. Rapeseed plants performed better in terms of seed yield (2.27 t ha^−1^) and oil yield (1.11 t ha^−1^) (Fig. 3), on average. Regarding crop residue yield, Ethiopian mustard had the highest average performance (5.49 t ha^−1^) over study years (Fig. 3).

### 3.4 Fatty acid profile, qualitative and quantitative characteristics of PVO and DSM

The year did not have any significant effect on PVO fatty acid profiles and quali-quantitative characteristics of PVO and DSM of the two oilseed crops (Table 3). Any significant variation was produced by replicate factor over the two years.

**Table 3:** Fatty acid profile of pure vegetable oil extrated from seed and defatted seed meal during two consecutive growing seasons. Average ± standard deviation values are shown (*n = 3*).

**Species**		**Palmitic acid (% d.m.)**	**Stearic acid (% d.m.)**	**Oleic acid (% d.m.)**	**Linoleic acid (% d.m.)**	**Linolenic acid (% d.m.)**	**Arachidic acid (% d.m.)**	**Gadoleic acid (% d.m.)**	**Cis 11 14-eicosadienoic acid (% d.m.)**	**Behenic acid (% d.m.)**	**Erucic acid (% d.m.)**	**Lignoceric acid (% d.m.)**	**Nervonic acid (% d.m.)**	**Other acids (% d.m.)**
*B. napus PR46W14*														
Seed		4.70 ± 0.06	1.73 ± 0.10	59.12 ± 0.59	19.23 ± 0.08	8.90 ± 0.13	0.52 ± 0.04	1.77 ± 0.19	–	0.32 ± 0.04	3.27 ± 0.60	–	0.23 ± 0.05	0.10 ± 0.00
*Significance*														
	Year	n.s.												
	Replicate	n.s.												
Defatted seed meal		4.32 ± 0.04	1.65 ± 0.05	57.85 ± 0.23	18.45 ± 0.12	12.07 ± 0.14	0.77 ± 0.08	8.12 ± 0.08	0.85 ± 0.05	0.73 ± 0.05	4.95 ± 0.37	–	1.43 ± 0.05	0.87 ± 0.27
*Significance*														
	Year	n.s.												
	Replicate	n.s.												
*B. carinata ISCI 7*														
Seed		3.15 ± 0.05	1.35 ± 0.05	12.32 ± 0.60	15.68 ± 0.16	12.43 ± 0.12	0.87 ± 0.05	9.10 ± 0.06	1.10 ± 0.00	0.70 ± 0.00	39.18 ± 0.51	0.52 ± 0.08	1.78 ± 0.04	1.72 ± 0.10
*Significance*														
	Year	n.s.												
	Replicate	n.s.												
Defatted seed meal		3.42 ± 0.12	1.38 ± 0.13	12.65 ± 1.00	16.43 ± 0.14	12.43 ± 0.12	0.85 ± 0.05	9.02 ± 0.16	1.10 ± 0.00	0.68 ± 0.04	38.07 ± 0.77	0.48 ± 0.04	1.78 ± 0.09	1.68 ± 0.28
*Significance*														
	Year	n.s.												
	Replicate	n.s.												

d.m.: dry matter, value expressed on dry basis.

n.s.: not significant.

Different fatty acid profiles were found mainly in relation to oleic and erucic acid content. Rapeseed PR46W14 had the greatest percentage of the oleic acid fraction (59.12 ± 0.59) and the lowest percentage of the erucic acid fraction (3.27 ± 0.60) over dry matter. Conversely, Ethiopian mustard ISCI 7 showed the greatest percentage of the erucic acid fraction (39.18 ± 0.51) and the lowest percentage of the oleic acid fraction (12.32 ± 0.60) over dry matter. These differences were mainly due to the fact that rapeseed PR46W14 was “double-zero” hybrid. Concerning unsaturated fatty acids, a high linoleic and linolenic acid fraction in % over dry matter were determined in both the species. The fatty acid profiles of DSMs resulted very similar to those of PVOs for the two crops (Table 3).

With regards to the qualitative and quantitative characteristics of PVOs and DSMs (Table 4), elementary analysis of seeds and DSMs showed that the oil content of the seeds as a % over dry matter was found to be higher than that of DSMs. The two species had high values of seed oil content (45.73 % DM for rapeseed and 44.88 % DM for Ethiopian mustard), on average. Regarding protein content, it was observed that Ethiopian mustard ISCI 7 had the highest average content in the seeds over the two growing seasons. In general, it was observed that seeds and DSMs had the highest percentage content for C and the lowest for N. The C–H–N fractions were the same in relative proportions for both raw materials in both the species. The ash content as a % over dry matter was found to be highest in the seeds and DSMs of Ethiopian mustard ISCI 7, while the GCV values of DSMs were over 4500 kcal kg^−1^ for both the crops.

**Table 4:** Elementary analysis of seed and defatted seed meal during two consecutive growing seasons. Average ± standard deviation values are shown (*n = 3*).

				**C–H–N (% d.m.)**				
**Species**		**Oil content (% d.m.)**	**Protein (% d.m.)**	**C**	**H**	**N**	**Ash (% d.m.)**	**GCV (Kcal kg^-1^)**
*B. napus PR46W14*								
Seed		45.73 ± 0.60	19.00 ± 0.26	61.32 ± 0.34	8.90 ± 0.22	3.10 ± 0.06	3.93 ± 0.12	–
*Significance*								
	Year	n.s.						
	Replicate	n.s.						
Defatted seed meal		14.88 ± 0.55	34.52 ± 0.39	49.65 ± 0.46	7.52 ± 0.10	5.35 ± 0.08	6.82 ± 0.08	4578.67 ± 154.62
*Significance*								
	Year	n.s.						
	Replicate	n.s.						
*B. carinata ISCI 7*								
Seed		44.88 ± 2.04	22.83 ± 0.40	60.20 ± 0.13	8.85 ± 0.19	3.62 ± 0.10	4.72 ± 0.08	–
*Significance*								
	Year	n.s.						
	Replicate	n.s.						
Defatted seed meal		32.13 ± 0.23	28.37 ± 0.39	55.75 ± 0.19	8.27 ± 0.08	4.57 ± 0.08	7.60 ± 0.13	5422.00 ± 16.53
*Significance*								
	Year	n.s.						
	Replicate	n.s.						

d.m.: dry matter, value expressed on dry basis; C: carbon; H: hydrogen; N: nitrogen; GCV: gross calorific value.

n.s.: not significant.

The glucosinolate contents of seeds (Table 5) ranged from 24.72 μmol g^−1^ (rapeseed PR46W14) to 84.29 (Ethiopian mustard ISCI 7) μmoles g^−1^ over dry matter. The two species had different GL content regarding seed and DSM. Particularly, glucosinalbin was the main GL for rapeseed while sinigrin was the predominant GL for Ethiopian mustard.

**Table 5:** Glucosinolates content of seed and defatted seed meal during two consecutive growing seasons. Average ± standard deviation values are shown (*n = 3*).

**Species**		**PRO (% d.m.)**	**SIN (% d.m.)**	**GAL (% d.m.)**	**SNB (% d.m.)**	**GNA (% d.m.)**	**4-OHGBS (% d.m.)**	**GBN (% d.m.)**	**GBS (% d.m.)**	**4-OMGBS (% d.m.)**	**NGBS (% d.m.)**	**Glucosinolates content (μmol g^-1^)**	
												**(on a.r.)**	**(on d.m.)**
*B. napus* PR46W14													
Seed		4.69 ± 0.01	1.27 ± 0.23	0.33 ± 0.01	11.46 ± 2.24	1.64 ± 0.15	2.49 ± 0.10	0.46 ± 0.01	0.11 ± 0.01	0.07 ± 0.01	0.29 ± 0.07	23.01 ± 2.14	24.72 ± 2.39
*Significance*													
	Year	n.s.											
	Replicate	n.s.											
Defatted seed meal		4.72 ± 3.67	22.42 ± 23.89	–	15.31 ± 4.78	1.64 ± 1.11	3.38 ± 0.10	0.78 ± 0.02	0.21 ± 0.04	0.12 ± 0.02	0.45 ± 0.15	48.89 ± 13.43	66.02 ± 2.94
*Significance*													
	Year	n.s.											
	Replicate	n.s.											
*B. carinata ISCI 7*													
Seed		–	77.62 ± 1.69	–	2.95 ± 0.85	–	0.82 ± 0.18	–	–	–	–	79.73 ± 0.66	84.29 ± 0.94
*Significance*													
	Year	n.s.											
	Replicate	n.s.											
Defatted seed meal		–	96.98 ± 1.09	–	7.37 ± 0.13	–	5.74 ± 0.20	–	–	–	–	113.61 ± 16.08	104.93 ± 0.25
*Significance*													
	Year	n.s.											
	Replicate	n.s.											

a.r.: as received by the analyst, value expressed on wet basis; d.m.: dry matter, value expressed on dry basis; PRO: progoitrin; SIN: sinigrin; GAL: glucoalyssin; SNB: glucosinalbin; GNA: gluconapin; 4-OHGBS: 4-hydroxy-glucobrassicin; GBN: glucobrassicanapin; GBS: glucobrassicin; 4-OMGBS: 4- metoxy-glucobrassicin; NGBS: neoglucobrassicin.

n.s.: not significant.

### 3.5 Chemical-physical characteristics of PVOs

Regarding the influence of the main factors on chemical-physical characteristics of PVOs, it was found that year and replicate did not determine significant differences for any parameter (Table 6).

**Table 6:** Chemical-physical characteristics of pure vegetable oil during two consecutive growing seasons. Average ± standard deviation values are shown (*n = 3*).

**Species**		**Oil mass volume (kg m^-3^)**	**Oil viscosity at 40 °C (mm2 s^-1^)**	**Iodine number (g I 100 g^-1^)**	**Oil acidity (% as oleic acid)**
*B. napus PR46W14*		911.79 ± 0.78	28.47 ± 0.26	111.01 ± 0.14	1.46 ± 0.01
*Significance*					
	Year	n.s.			
	Replicate	n.s.			
*B. carinata ISCI 7*		910.54 ± 0.79	36.39 ± 0.10	111.16 ± 0.13	0.26 ± 0.01
*Significance*					
	Year	n.s.			
	Replicate	n.s.			

n.s.: not significant.

On average, the oil mass volumes of the two species were found to be similar. Ethiopian mustard ISCI 7 showed the highest average oil viscosity at 40 °C (36.39 mm^2^ s^−1^) caused by a higher erucic acid percentage content. The iodine value was found to be highly similar between the species, and oil acidity was greatest in the rapeseed PR46W14 (1.46 % as oleic acid), on average.

### 3.6 Chemical-physical characteristics of crop residues

The two random factors did not significantly affect the chemical-physical parameters of crop residues (Table 7).

**Table 7:** Chemical-physical characteristics of crop residue during two consecutive growing seasons. Average ± standard deviation values are shown (*n = 3*).

**Species**		**Moisture (% d.m.)**	**Ash (% d.m.)**	**Fiber (% d.m.)**			**C–H–N (% d.m.)**			**Glucides (% d.m.)**	**GCV (MJ kg^-1^ d.m.)**
				**NDF**	**ADF**	**ADL**	**C**	**H**	**N**		
*B. napus PR46W14*		5.73 ± 0.09	5.94 ± 0.37	76.51 ± 0.78	65.60 ± 0.39	10.79 ± 0.43	44.91 ± 0.24	6.68 ± 0.05	0.51 ± 0.01	6.74 ± 0.13	15.78 ± 0.05
*Significance*											
	Year	n.s.									
	Replicate	n.s.									
*B. carinata ISCI 7*		5.52 ± 0.29	5.95 ± 0.21	75.60 ± 0.30	63.29 ± 1.43	11.18 ± 0.27	45.58 ± 0.47	6.61 ± 0.03	0.56 ± 0.07	8.16 ± 0.24	15.38 ± 0.18
*Significance*											
	Year	n.s.									
	Replicate	n.s.									

d.m.: dry matter, value expressed on dry basis; NDF: neutral detergent fiber; ADF: acid detergent fiber; ADL: acid detergent lignin; C: carbon; H: hydrogen; N: nitrogen; GCV: gross calorific value.

n.s.: not significant.

Similar moisture contents were observed in the two species on average. During the test period, moisture content was below 6.00 % d.m. for both species. Crop residue ash content was 5.94 % for rapeseed PR46W14 and 5.95 % d.m. for Ethiopian mustard ISCI 7, on average. The NDF, ADF and ADL contents of crop residues were extremely similar between the species and low variation was observed between the two growing seasons. In particular, NDF was the main fiber for rapeseed and Ethiopian mustard at over 75% d.m. while ADL was the least predominant fiber for both species, below 11% d.m. In both the species, the carbon was the most abundant element for crop residues while nitrogen was the least as a percentage of dry matter. Crop residues of Ethiopian mustard ISCI 7 had the greatest glucide content (8.16 % DM), on average. Regarding the energetic properties of crop residues, the gross calorific values were found to be higher than 15.0 MJ kg^−1^ d.m. for both crops, on average.

### 3.7 Calculation of the incentives for PVOs in different years and cash flow analysis

The identification of the type of power plant and biofuel were crucial in order to calculate the incentives. In accordance with Italian Decrees July 06, 2012 and June 23, 2016, incentives are intended for new energy plants, reactivations, complete reconstructions and upgrades and vary according to size and source. PVOs obtained from the two *Brassica* species are added among sustainable bioliquids.

In 2012–2014, the base feed-in incentive for sustainable bioliquids provided by Italian Decree July 06, 2012 was calculated to be 121 € MW h^−1^, considering an expected service life of the CHP plant of 20 years starting from January 01, 2013. For a total of 55.21 kW, the overall base inventive was 6.70 € h^−1^. A high-efficiency cogeneration premium (40 € MW h^−1^) was related to net energy production in accordance with this Decree; therefore, the all-inclusive incentive was equal to 8.91 € h^−1^.

In 2023, the base feed-in incentive for sustainable bioliquids provided by Italian Decree June 23, 2016 was found to be 60 € MW h^−1^, considering an expected service life of the CHP plant of 20 years starting from December 31, 2017. For a total of 55.21 kW, the overall base incentive was calculated as 3.30 € h^−1^. According to this Decree, with reference to CHP plants operating before June 30, 2017 only, a high-efficiency cogeneration premium (40 € MW h^−1^) was related to net energy production. Consequently, in 2023, the all-inclusive feed-in incentive was equal to the base feed-in incentive without any premium. When comparing the two periods, it is evident that the incentives for PVO production using a CHP plant were more attractive in 2012–2014 than 2023.

The cash flow results are shown in Supplementary Table 1. The electricity production was expected to be equal over the 20-year period. This was due to the same all-inclusive feed-in incentive provided by Italian Decree July 06, 2012 and net energy produced by the combined heat and power plant in the same period. The thermal energy production was calculated to be increased depending on the operating of the CHP plant. The sale price of DSM varied over the years based on the estimated prices of raw materials. The production costs of PVOs increased over the years due to the increasing estimated market prices of the oil. The ordinary maintenance costs of the CHP plant increased year-by-year depending on the operating age of the machine. However, the increase of these costs was not estimated to be very high. With regards to extra-ordinary maintenance costs, they were calculated every two years following the guidelines of the maintenance manual. They were found to be very similar.

**Fig. 4:**
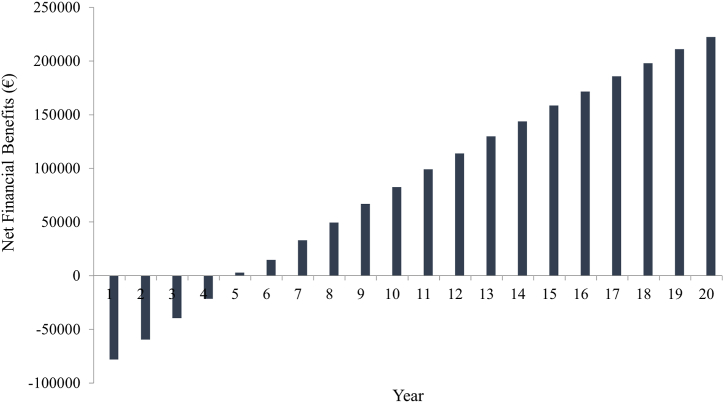
Cash flow trends considering the service life of the CHP plant.

Fig. 4 shows the results of cash flow taking into consideration a CHP plant's service life of 20 years with reference to 2012–2014 period. Trend analysis results highlights potential economic interest to farmers.

## 4 Discussion

In this study, rapeseed and Ethiopian mustard varieties were well suited to the soil and weather conditions in the experimental site and confirmed their potential as oilseed crops for cultivation in the Southern Mediterranean region. Their growth cycle differed in length and the crops accumulated different GDDs over the two growing seasons. In both years, early sowing was carried out in autumn to save on the amount of irrigation water supplied to the crops to satisfy their needs. At rosette growth stage, the crops showed a remarkable degree of resilience in the face of lower winter temperatures, they were able to exploit the autumn/winter rainfall at stem elongation stage and demonstrated good tolerance to higher spring/summer temperatures at fruit ripening and senescence stages. This behaviour was well explained by other authors who conducted comparative studies on oilseed crops under different climate conditions [[52], [53], [54]].

Regardless of input levels, over the two-year period, rapeseed showed constantly higher seed and oil yields than Ethiopian mustard and this was confirmed by literature. In Italy, Zanetti et al. [14], when comparing varieties of the two crops under different input management systems, found that rapeseed varieties had greatest seed yield and oil content due to more stable behaviour over the years. In a comparative study of rapeseed and Ethiopian mustard in different Italian areas, Del Gatto et al. [10] found that the seed yields of certain rapeseed cultivars varied greatly between locations, whilst, on average, productivity of Ethiopian mustard was lower due to evident variability in the environments. In our study, for example, seed yields of the two crops, on average, were detected lower than those tested in Central and Northern Italian areas. This means that higher average temperatures and prolonged periods of drought experienced in the Sicilian area compared to other areas negatively affected plant growth, leading to reductions in seed yield in both species. However, a lower seed yield does not mean that the crops are not suited to the test environment, bearing in mind that the rusticity of rapeseed has been demonstrated by several studies [3,13,55].

In Sicily and other areas of the South Europe, the lack of industries for biodiesel processing and production has highlighted the importance of short agri-energy supply chains which would allow farmers to exploit all raw materials produced by oilseed crops such as PVO, DSM and crop residues. It is evident that the qualitative characteristics of the raw materials needs careful investigation to assess bioenergy levels produced by the organic materials in an agri-energy supply chain.

Regarding PVO, it is well-known that the fatty acid configuration can affect the properties of PVO [56]. In previous studies, it has been demonstrated that the resistance of oils to the oxidative breakdown lasts longer when they contain higher level of monounsaturated fatty acids with respect to level of polyunsaturated fatty acids [57,58]. In our study, PVOs extracted from rapeseed were characterized by a high oleic acid content whilst those extracted from Ethiopian mustard showed a high erucic acid content. PVO quality also depends on various chemical-physical properties, one of the most important being oil viscosity. Viscosity affects engine performance during energy cogeneration and determines the lubricant's film strength and efficiency in preventing friction between moving parts. In general, the main issue by using PVOs as fuel for diesel engines is the high viscosity; it can cause injector fouling during compression ignition and other problems with the engine [59,60]. In our study, to avoid this problem, during the energy cogeneration process, diesel fuel was exploited to start and stop engines. Furthermore, PVOs were entered the combustion chamber after being warmed to 80 °C in order to decrease oil viscosity and to avoid obstruction of the filters, in accordance with Ramkumar and Kirubakaran [61]. This strategy was adopted also in light of greater oil density and iodine values compared to diesel fuel. The heating value is an indicator of the amount of energy released per fuel quantity, and the GCV is one of the most important properties of a fuel [59]. In the present study, GCV values obtained from DSM were over 4500 kcal kg^−1^ for both species. Higher GCV values have been estimated for diesel fuel, therefore, it is not possible to make any comparison with the GCV values of PVOs.

DSM is mainly derived from seed defatting procedures and it is largely-used for bioenergy and green chemistry [62]. Rapeseed DSM consists of proteins with a well-balanced amino acid composition [63] and provides a good protein source to address the increasing global demand for protein [64]. Some authors [65,66] report that DSM obtained from rapeseed “double-zero” varieties, can be an excellent feed component in monogastric animal nutrition and a valid alternative to soybean meal due to a low content of antinutritional factors and high crude protein content. Based on a higher C level of DSMs than N, the soil incorporation of DSMs in the long-term period could increase and improve the carbon stock, thus improving the soil fertility. This represents a sustainable practice that could increase the organic matter content in soils with a relatively lower C content such as sandy soils. The effect of the use of Ethiopian mustard DSM as a soil amendment was well described by Monaci et al. [67].

Crop residue is an important co-product of agriculture and its agronomic valorisation provides various benefits for farmers. It is a well-known agricultural practice to incorporate crop residues into soil to provide organic carbon and nitrogen inputs, thus ensuring soil fertility is at least maintained or improved [68]. In our study, crop residues of the two *Brassica* species were found to contain high levels of carbon, low levels of nitrogen and a good fiber and glucide content. It has been reported that the application of *Brassica* residues on soils can improve soil health and crop productivity in the medium/long term [69,70], reducing the current use of mineral and organic fertilisers on farm. Regarding biomass productivity, by comparing rapeseed and Ethiopian mustard varieties, crop residue yields were higher in Ethiopian mustard, which proves itself as an interesting oilseed crop for biomass production [71]. In a short agri-energy supply chain, the exploitation of crop residues for energy purposes mainly depends on residue availability in the long-term and energy properties of the material [72]. Evidence highlights that the crop rotation system used affects the availability of agricultural residues [73]. Therefore, the present short agri-energy supply chain needs to include two or more farms so that, according to the rotation systems adopted, the species can be present each year. Monforti et al. [72] affirmed that crop residues can be viewed as a valuable resource to generate electricity, heating and cooling as well. Furthermore, oilseed crop residues can be converted directly into second-generation cellulosic biofuels without impacting on land use [74]. It is clear that a preliminary evaluation of the energetic characteristics of crop residues is required, however, this affects the performance of the power plant. In our study, rapeseed and Ethiopian mustard crop residues showed relatively high average moisture and ash contents. These values were similar to those found by Duca et al. [22] who assessed the qualitative characteristics of residues obtaining from biodiesel chains that included oilseed crops in the Mediterranean region. Taking into consideration the qualitative aspects, however, the high ash content of residues can be problematic for energy uses due to the fact it reduces combustion efficiency [75]. In addition, the relatively high N values could increase emissions in terms of NO_x_ during combustion [22]. The higher heating values of Ethiopian mustard and rapeseed crops were found to be similar to those of cotton (*Gossypium* spp.) maize (*Zea mays* L.), sunflower and sorghum (*Sorghum bicolor* (L.) Moench) but lower than various forest residues and fossil feedstocks [76]. Furthermore, the high C/N ratio of residues makes them unsuited for use in anaerobic digestion to ensure efficient biogas production [77]. However alternative solutions could be pursued in order to increase the value of these materials. For example, the mixture of *Brassica* residues with other agricultural and forest residues could improve the ash-residue properties and develop new ligno-cellulosic products of high-energy value [22,78,79].

In the present study a pilot combined heat and power plant operating on PVOs was exploited to produce bioenergy directly on-farm. The simultaneous production of electricity and thermal energy through the exploitation of PVOs makes the CHP system an economic/energetic opportunity for farmers. Indeed, the electricity could be either consumed directly on farm or sold to the Electrical Market Operator through incentives schemes, while thermal energy could be exploited to meet the farm's heat demand, for example. In this study, the examination of cash flows allowed us to determine a profitable net present value and an expected 5-year investment payback period. In a short agri-energy supply chain, these benefits can be achieved when two main conditions are met: long-term legislation which allows farmers a return on capital investments and number of farms within rural districts which direct part of the agricultural production from food crops (crops with a positive energy/environmental balance). However, energy policies tend to change over time depending on different factors and often do not guarantee the availability of incentives in the long-term. Furthermore, farmers change their crop planning in the long-term, depending on market opportunities and may reduce the production of PVOs in rural districts. From a supply chain management perspective, it is necessary to guarantee suitable collaboration tools between the various stakeholders in the agri-energy chain, to ensure efficient functioning within a framework of stable rules. These considerations play an important part in the agri-energy context: a farm that recognizes the added value, not only from an economic perspective, deriving from the choice of an energy production and distribution model based on short agri-energy supply chain, must necessarily ensure the traceability and sustainability of its production. On similar assumptions, the farm should benefit of a special incentive regime in the long-term.

## 5 Conclusions

This study carried out in a semiarid Mediterranean environment confirms that Ethiopian mustard and rapeseed are two promising oilseed crops which can encourage yield diversification on farms. Both oilseed crops adapted well to Sicilian climate conditions and provided significant yields. The evaluation of their productivity demonstrated significant differences between the crops in the same agronomic and environmental conditions. Rapeseed performed better in terms of oil and seed yields while Ethiopian mustard had the highest crop residue yield. These results represent a valid support for farmers to pursue multifunctional agriculture and valorise the agricultural feedstocks. In a short agri-energy supply chain, the exploitation of all raw materials produced by *Brassica* crops such as PVO, defatted seed meal and crop residue appears to be fundamental to optimize the contribution of agriculture to the production of bioenergy, according to European energy directives. PVO can be self-consumed on-farm or sold externally and allows farmers to become bioenergy producers. DSM can be used in various sectors. The *Brassica* crop residues could be mixed with other agricultural and forest residues to obtain products of high energy value.

In this study, a model based on cash-flow permitted to assess the economic aspects of the CHP plant. It highlighted relevant economic benefits to farmers during the CHP plant's service life but, at the same time, the need to ensure an appropriate government incentives in a long-term period to exploit the PVO as a biofuel.

## Funding statement

This study was supported by ANDROMEDA research project funded by the 10.13039/501100009869Sicilian Regional Ministry of Agricultural and Food Resources (G66D1100500009).

## Data availability statement

Data will be made available on request.

## CRediT authorship contribution statement

**Mario Licata:** Writing – original draft, Writing – review & editing, Funding acquisition, Conceptualization. **Davide Farruggia:** Writing – review & editing, Software, Methodology, Investigation, Data curation. **Giuseppe Di Miceli:** Writing – original draft, Resources, Methodology, Formal analysis. **Francesco Salamone:** Writing – original draft, Software, Investigation, Data curation. **Nicolò Iacuzzi:** Writing – original draft, Visualization, Validation, Data curation. **Teresa Tuttolomondo:** Writing – original draft, Supervision, Investigation, Funding acquisition.

## Declaration of competing interest

The authors declare that they have no known competing financial interests or personal relationships that could have appeared to influence the work reported in this paper.
